# Predicting postoperative coronal alignment in medial unicompartmental knee arthroplasty using the arithmetic hip-knee-ankle angle

**DOI:** 10.1186/s42836-025-00326-x

**Published:** 2025-08-06

**Authors:** Chengyuan Ma, Zifan Luo, Guanghui Zhao, Jianbing Ma, Jianpeng Wang

**Affiliations:** Department of Joint Surgery, Honghui Hospital, Xi’an Jiaotong University, No.555 East Youyi Road, Xi’an, 710054 Shaanxi China

**Keywords:** Unicompartmental knee arthroplasty, Coronal alignment, Arithmetic hip-knee-ankle angle

## Abstract

**Background:**

Proper coronal alignment plays a critical role in the effectiveness of medial unicompartmental knee arthroplasty (UKA). This research seeks to explore the connection between the arithmetic hip-knee-ankle angle (aHKA) and the actual postoperative (postop) HKA angle after undergoing medial UKA.

**Methods:**

A retrospective analysis was conducted on individuals who received medial UKA at a specialized orthopedic hospital between January 1, 2024, and July 31, 2024. The aHKA was determined using the formula: medial proximal tibial angle (MPTA) minus lateral distal femoral angle (LDFA), plus 180°. The relationships between the postop HKA angle and the aHKA, MPTA, and LDFA were analyzed. Patients were further divided into three categories based on their postop HKA angle: greater than 180°, between 175° and 180°, and less than or equal to 175°. These groups were then compared in terms of aHKA, LDFA, MPTA, and preoperative HKA angle.

**Results:**

A total of 242 patients (254 knees) were included in this study. The postop HKA was nearly equal to the preoperative aHKA (176.09° ± 2.86° vs. 176.23° ± 3.15°). Statistical analysis revealed a positive association between aHKA and postop HKA angle (R^2^ = 0.4595, *P* < 0.05), as well as between MPTA and postop HKA angle (R^2^ = 0.2072, *P* < 0.05). Conversely, a negative correlation was identified between LDFA and postop HKA angle (R^2^ = 0.2448, *P* < 0.05). These patterns held true for both fixed-bearing and mobile-bearing UKA prostheses. Notable differences among the three HKA groups were found regarding aHKA, MPTA, LDFA, and preoperative HKA angle (*P* < 0.05).

**Conclusion:**

The findings indicate that aHKA has a strong relationship with the postop HKA angle, suggesting its potential as an effective predictor for postop coronal alignment after medial UKA, irrespective of the type of prosthesis used.

## Background

Unicompartmental knee arthroplasty (UKA) serves as an effective treatment for individuals suffering from medial compartment osteoarthritis (OA), providing advantages such as less surgical trauma and the retention of healthy joint areas and cruciate ligaments [1–4]. Research had indicated that UKA demonstrates a 10-year survival rate between 91.4% and 98% [5, 6]. Nevertheless, despite these favorable outcomes, implant failure can still occur. According to the 15th Annual National Joint Replacement Report covering England, Wales, and Northern Ireland, the cumulative revision rate for UKA at 14 years reaches 16.9% [7]. Furthermore, a comprehensive review incorporating data from six national registries and multiple clinical investigations reported a 10-year revision rate of 16.5% for UKA [8]. These statistics highlight the importance of identifying and addressing factors associated with UKA failures to lower revision rates and improve long-term implant durability.

Earlier research had stressed the significance of coronal alignment in achieving successful UKA outcomes. Utilizing a prospective institutional database comprising 3351 cases of medial fixed-bearing UKA, Slaven et al. [9, 10] identified 37 revisions due to lateral compartment OA and 61 revisions attributed to aseptic loosening. In the group revised for lateral compartment OA, the HKA angle was significantly more valgus compared to the control group (0.3° ± 3.6° valgus vs. 4.4° ± 2.6° varus, *P* < 0.001). Conversely, in the aseptic loosening group, the HKA angle was significantly more varus (6.1° ± 3.1° varus vs. 4.0° ± 2.7° varus, *P* < 0.001). These findings suggest that maintaining optimal postoperative (postop) alignment—avoiding excessive varus or valgus—is crucial. However, there is currently limited research focused on identifying preoperative indicators that can accurately predict postop HKA angles. Recently, a novel morphological evaluation technique called the arithmetic hip-knee-ankle angle (aHKA) has attracted attention for its potential in assessing lower limb anatomy [11]. Unlike traditional methods, aHKA relies exclusively on bony anatomical landmarks and does not depend on the femorotibial relationship, making it unaffected by joint space narrowing. Additionally, this method has been validated across large populations and remains consistent regardless of whether imaging is performed while standing or lying down. As a result, aHKA is expected to minimize inter-patient measurement variability, offering a promising approach for preoperative planning in UKA [11, 12].

This study aims to explore the connection between preoperative aHKA measurements and postop coronal alignment following medial UKA. We propose that a significant association exists between preoperative aHKA values and the resulting postop HKA angle after medial UKA.

## Methods

This retrospective study was carried out at a specialized orthopedic medical center, focusing on individuals who received UKA from January 1, 2024, to July 31, 2024. The clinical indications for performing UKA were defined as follows: (1) presence of anteromedial osteoarthritis; (2) intact ligaments around the knee joint; (3) flexion contracture not exceeding 15° with maintained range of motion; and (4) correctable varus deformity less than 15° [13, 14]. The selection criteria for this research encompassed: (1) patients undergoing UKA due to varus deformity associated with osteoarthritis, and (2) availability of standardized weight-bearing full-leg radiographic images taken both before and after surgery. Cases involving radiographs of insufficient quality for accurate evaluation were excluded from the study. Prior to May 1, 2024, the sole UKA implant available was the LINK® Sled fixed-bearing prosthesis manufactured by Waldemar Link GmbH & Co.KG in Hamburg, Germany. However, during the study period, only mobile-bearing UKA prostheses supplied by Chunlizhengda Medical Instruments Co., Ltd., based in Beijing, China, were accessible, as fixed-bearing prostheses were unavailable due to regulatory restrictions. All surgical procedures were performed by a team of surgeons utilizing conventional instrumentation techniques. Ethical approval for the study was granted by the hospital’s institutional review board (reference number: 202409003). Radiographic data were retrieved from the hospital imaging system following verbal consent obtained via telephone interviews with the patients.

### Surgical technique

In the UKA procedure, the surgical objective was to restore the knee joint’s alignment to its pre-arthritic state, guided by the principle of surface replacement, regardless of whether a fixed-bearing or mobile-bearing prosthesis was used. Surgical incisions were initiated at the anatomical landmark formed by the junction of the upper edge of the patella and the inner margin, extending to the inner side of the tibial tuberosity, with a standard length of 8–10 cm. Tibial plateau osteotomy was performed using extramedullary positioning, with the osteotomy line oriented perpendicular to the tibial mechanical axis. For fixed-bearing prostheses, the distal femur was manually prepared, with femoral prosthesis positioning based on tibial plateau gap-balance. In contrast, for mobile-bearing prostheses, distal femoral osteotomy was performed using standardized intramedullary positioning. The long axis of the femoral prosthesis, irrespective of prosthesis type, was aligned to remain parallel to the long axis of the tibial prosthesis when the knee was flexed at 90°. The insert thickness selection for both prosthesis types was determined by two key criteria: ease of implantation during flexion and extension, and the ability of the medial gap to open by approximately 1 mm under external rotation stress. These considerations ensured optimal postop joint function and stability.

### Radiographic assessments

All patients underwent standardized weight-bearing long-leg radiographs before surgery. On the day after surgery, the same radiographic series was repeated when patients could straighten the knee joint and stand independently. These radiographs were acquired using the protocol described by Paley et al. [15]. In a standard weight-bearing long-leg radiograph, the fibular heads overlap the tibia by approximately one-third on both sides, the patella is positioned vertically and centrally relative to the knee joint, and the lesser trochanters on both sides exhibit similar morphology [16].

**Fig. 1:**
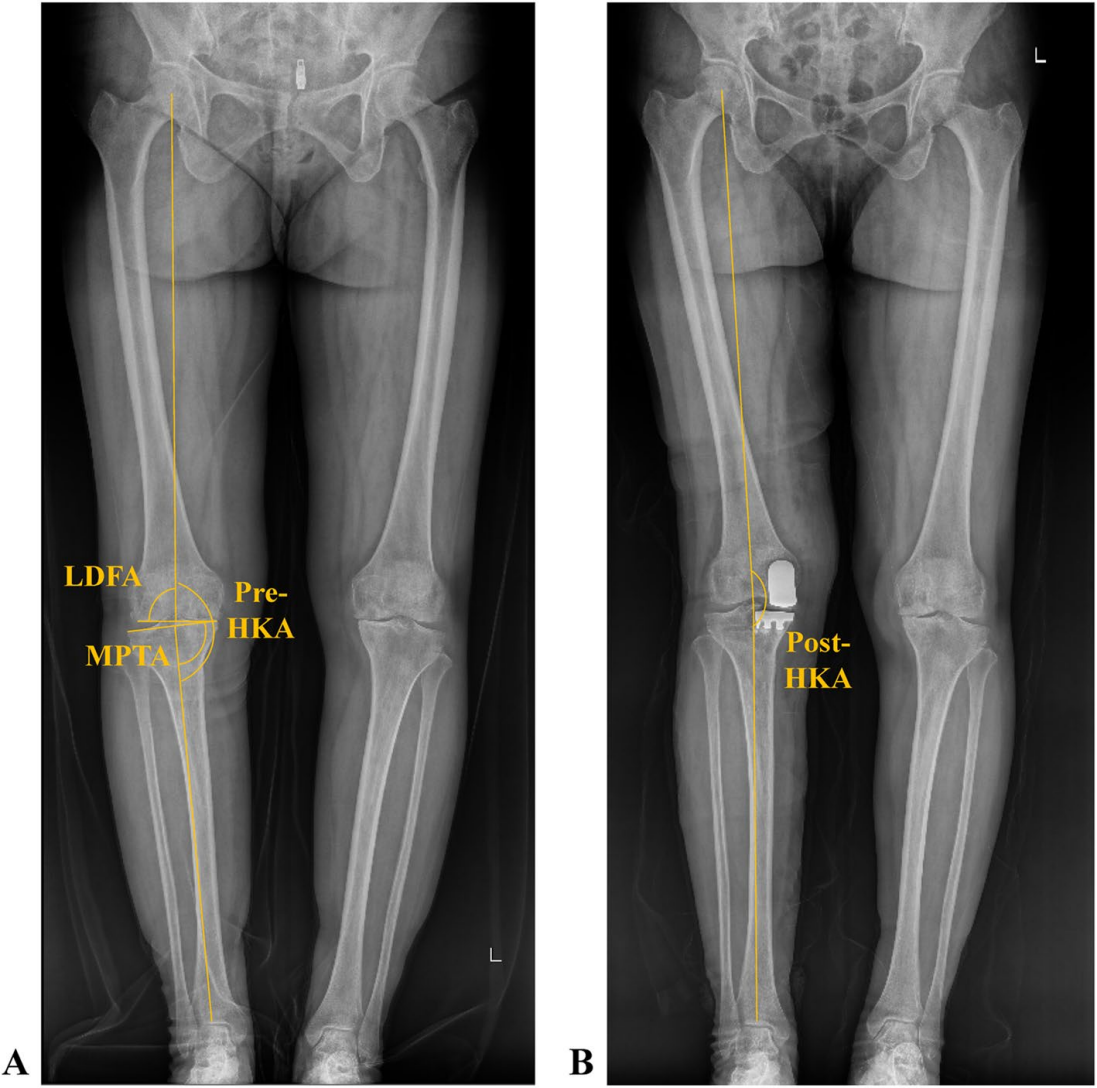
Measurement of different alignment parameters on the weight-bearing long-leg radiograph. Pre-HKA, preoperative hip-knee-ankle angle; post-HKA, postop hip-knee-ankle angle; LDFA, lateral distal femoral angle; MPTA, medial proximal tibial angle

Measurements of the preoperative HKA angle, MPTA, LDFA, and postop HKA angle were taken using the hospital’s imaging system (PACS) (Fig. 1). The HKA angle is defined as the angle between the tibial and femoral mechanical axes. The MPTA is the angle between the tangents to the medial and lateral tibial plateaus and the tibial mechanical axis. The LDFA is the angle between the tangents to the medial and lateral femoral condyles and the femoral mechanical axis [17–19]. The aHKA was calculated by subtracting the LDFA from the MPTA, as proposed by MacDessi et al. [12]. For this study, aHKA was defined as MPTA—LDFA + 180° to facilitate comparison with the postop HKA angle [20].

### Statistical analysis

Continuous variables, including age, preoperative HKA, LDFA, MPTA, aHKA, and postop HKA, were presented as means ± standard deviations (SD). Categorical variables, such as sex and side, were presented as frequencies and percentages (%). Sample size determination was performed using G*Power software (Version 3.1.9). Based on previous studies, a minimum sample size of 89 patients was required for single regression analysis, assuming an effect size (f^2^) of 0.15, a type I error (α) of 0.05, and a power (1 − β) of 0.95. To assess the intra-observer and inter-observer reliability of measurements for the LDFA and MPTA angles, two investigators independently evaluated the first ten patients twice. Intra-class correlation coefficients (ICCs) were calculated. For intra-observer reliability, ICCs were > 0.84 (range 0.84–0.97), and for inter-observer reliability, ICCs were > 0.83 (range 0.83–0.97) across all measurements.

**Fig. 2:**
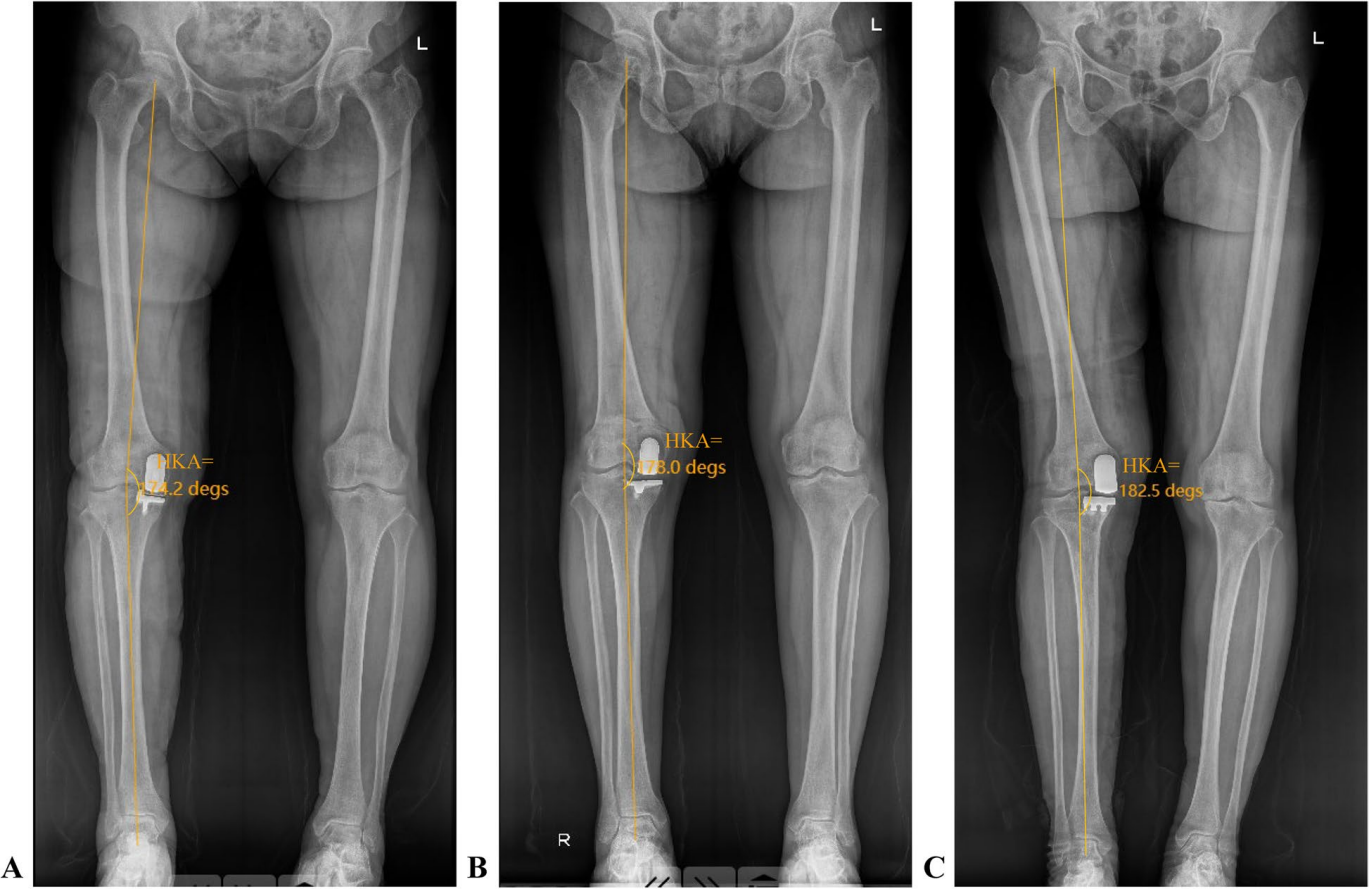
Three groups based on the patients’ postop HKA angle: **A**. group severe varus (HKA angle = 174.2° ≤ 175°); **B**. group mild varus (175° < HKA angle = 178° ≤ 180°); and **C**. group valgus (HKA angle = 182.5° > 180°)

Single regression analysis was conducted to investigate correlations between the postop HKA angle and the aHKA, LDFA, and MPTA, considering different prosthesis types. Patients were categorized into three groups based on their postop HKA angle (Fig. 2): Group Valgus (HKA angle > 180°), Group Mild Varus (175° < HKA angle ≤ 180°), and Group Severe Varus (HKA angle ≤ 175°). Differences in aHKA, LDFA, MPTA, and preoperative HKA angle among these groups were compared. Spearman correlation analyses examined the relationship between postop HKA and aHKA, LDFA, and MPTA. Differences among the three groups (Valgus, Mild Varus, Severe Varus) were assessed using one-way analysis of variance (ANOVA). Statistical analyses were performed using SPSS 25.0 (IBM, New York, USA), with a *P*-value < 0.05 (two-sided) considered statistically significant. Statistical images were plotted using GraphPad Prism (Version 9.5.1, Boston, USA).

## Results

This study included 254 knees from 242 patients. The mean patient age was 65.33 ± 6.77 years (range: 49–83 years), with 90 males (37.19%) and 152 females (62.81%). Surgery involved the left knee in 129 cases (50.79%) and the right knee in 125 cases (49.21%). Prosthesis types comprised 161 fixed-bearing (63.39%) and 93 mobile-bearing (36.61%). The mean aHKA was 176.23° ± 3.15°, and the mean postop HKA was 176.09° ± 2.86°. Other preoperative radiological parameters, including the LDFA and MPTA, are detailed in Table 1. Based on postop HKA, knees were classified as valgus (*n* = 16, 6.30%), mild varus (*n* = 157, 61.81%), or severe varus (*n* = 81, 31.88%).

**Table 1:** Basic characteristics

Variables	
Patients (knees)	242 (254)
Age (years)	65.33 ± 6.77
Sex	
Female	152 (62.81%)
Male	90 (37.19%)
Side of UKA	
Right	125 (49.21%)
Left	129 (50.79%)
Prothesis type	
Fixed-bearing	161 (63.39%)
Mobile-bearing	93 (36.61%)
Radiological parameters	
Preoperative HKA (°)	171.71 ± 3.22
MPTA (°)	86.27 ± 2.34
LDFA (°)	90.05 ± 2.17
aHKA (°)	176.23 ± 3.15
aHKA (°)	
≤ 175°	88 (34.65%)
175° < HKA ≤ 180°	141 (55.51%)
> 180°	25 (9.84%)
Postop HKA (°)	176.09 ± 2.86
≤ 175°	81 (31.89%)
175° < HKA ≤ 180°	157 (61.81%)
> 180°	16 (6.30%)

UKA, unicompartmental knee arthroplasty; HKA, hip-knee-ankle; MPTA, medial proximal tibial angle; LDFA, lateral distal femoral angle; aHKA, arithmetic hip-knee-ankle angle

**Fig. 3:**
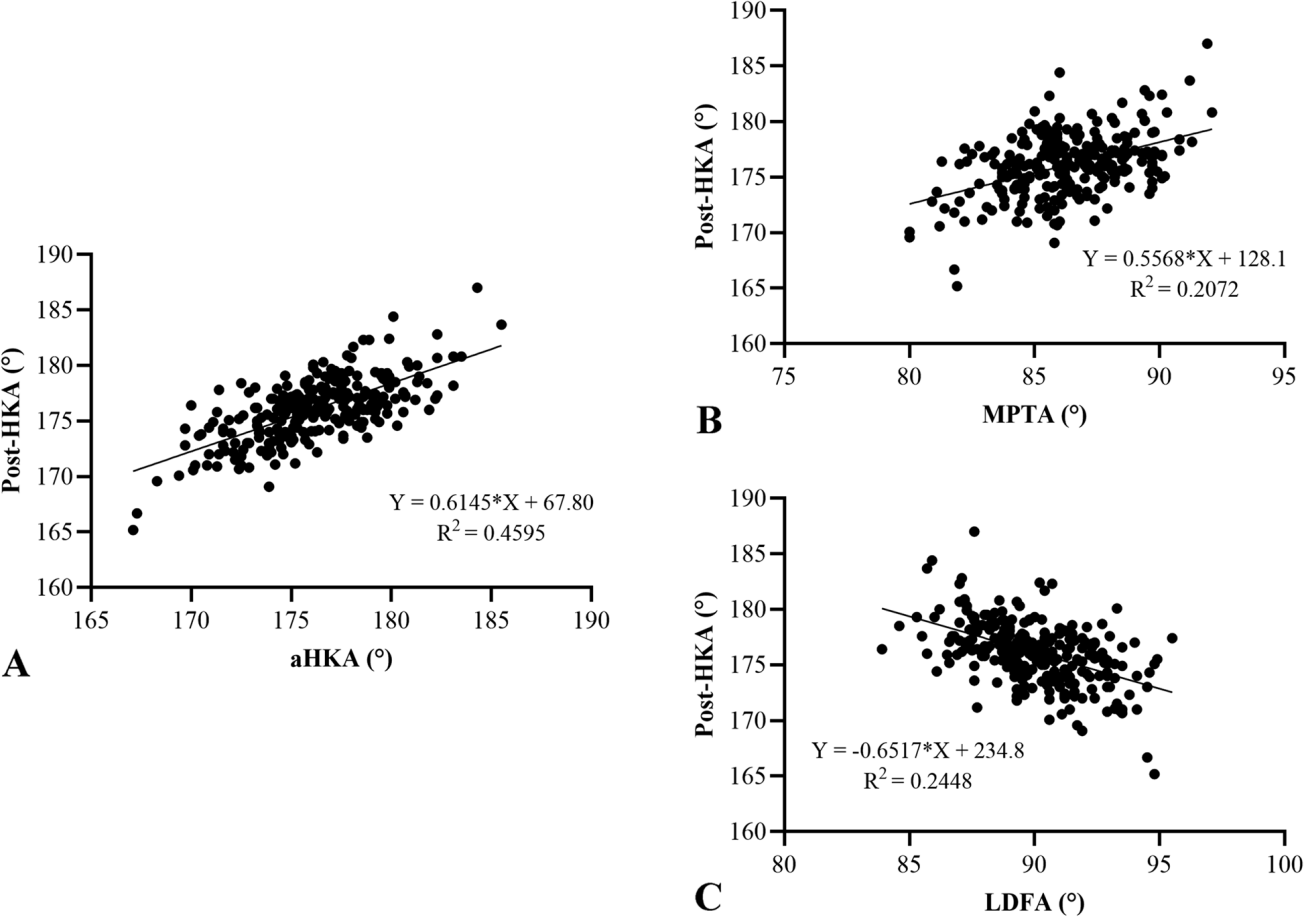
Correlation analysis between aHKA (**A**), MPTA (**B**), LDFA (**C**), and postop HKA angle. The aHKA and MPTA were positively correlated with the postop HKA angle. LDFA was negatively correlated with the postop HKA angle. HKA, hip-knee-ankle; MPTA, medial proximal tibial angle; LDFA, lateral distal femoral angle; aHKA, arithmetic hip-knee-ankle angle

**Fig. 4:**
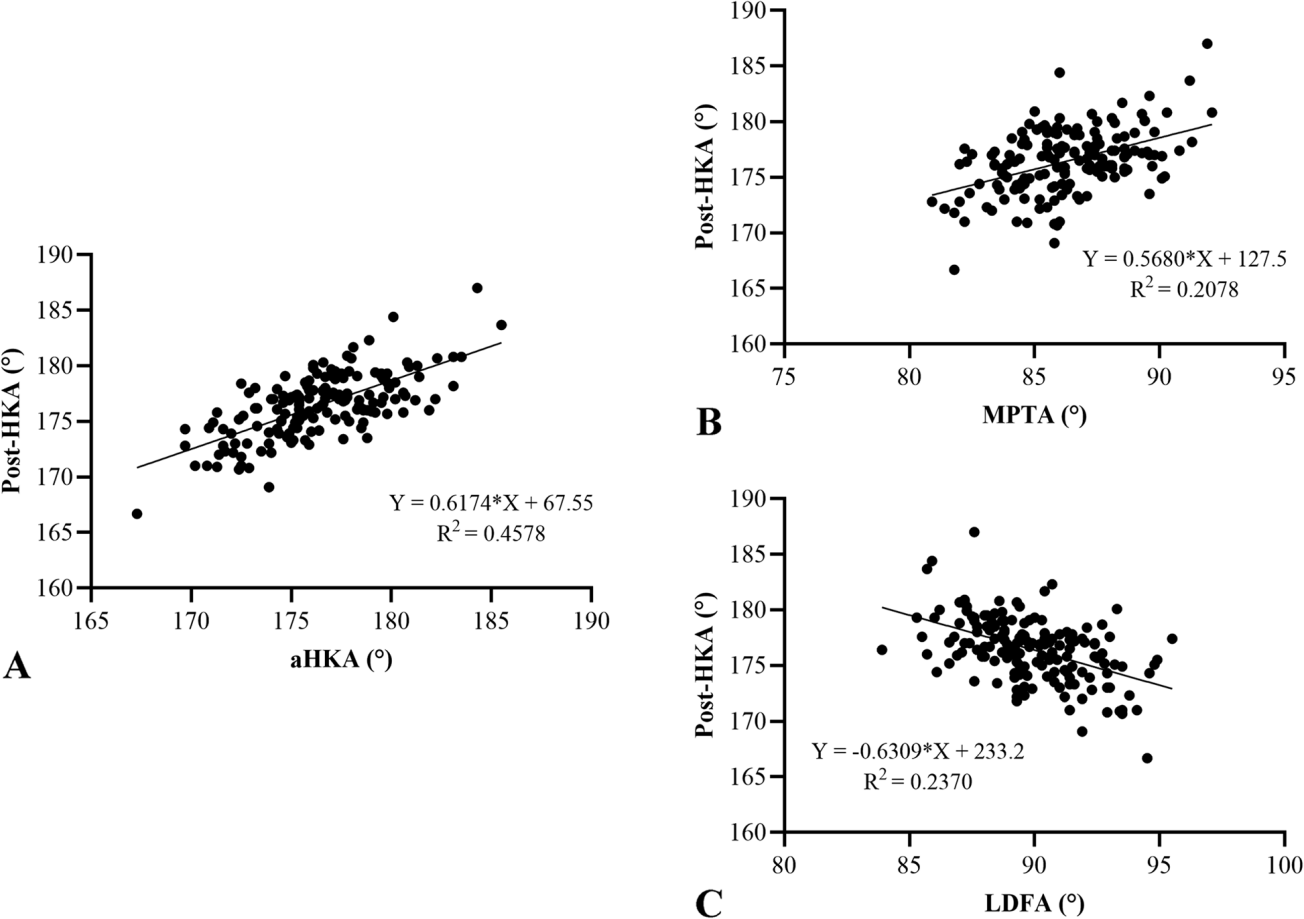
Correlation analysis between aHKA (**A**), MPTA (**B**), LDFA (**C**) and postop HKA angle in fixed-bearing UKA. The aHKA and MPTA was positively correlated with the postop HKA angle. LDFA was negatively correlated with the postop HKA angle. HKA, hip-knee-ankle; MPTA, medial proximal tibial angle; LDFA, lateral distal femoral angle; aHKA, arithmetic hip-knee-ankle angle

**Fig. 5:**
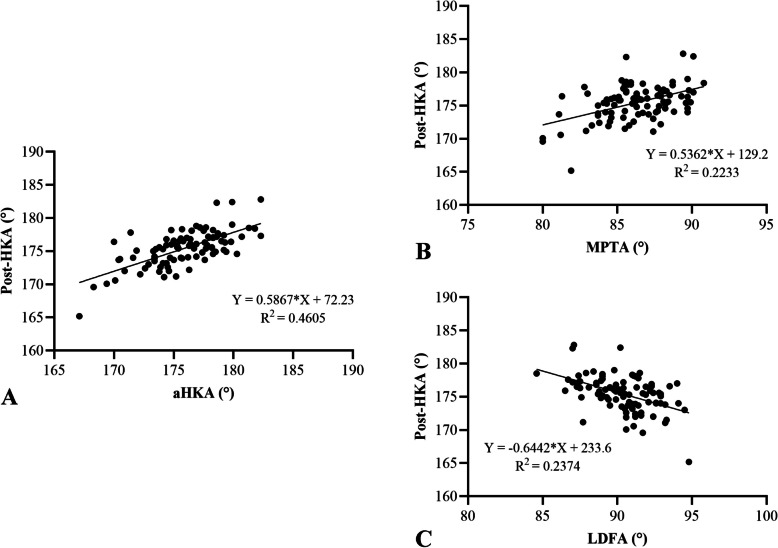
Correlation analysis between aHKA (A), MPTA (**B**), LDFA (**C**) and postop HKA angle in mobile-bearing UKA. The aHKA and MPTA was positively correlated with the postop HKA angle. LDFA was negatively correlated with the postop HKA angle. HKA, hip-knee-ankle; MPTA, medial proximal tibial angle; LDFA, lateral distal femoral angle; aHKA, arithmetic hip-knee-ankle angle

Spearman correlation analysis revealed significant positive correlations between postop HKA and both aHKA (R^2^ = 0.4595, *P* < 0.05) and MPTA (R^2^ = 0.2072, *P* < 0.05). A significant negative correlation was observed between postop HKA and LDFA (R^2^ = 0.2448, *P* < 0.05) (Fig. 3). Subgroup analyses by prosthesis type yielded consistent findings. For fixed-bearing prostheses, postop HKA correlated positively with aHKA (R^2^ = 0.4578, *P* < 0.05) and MPTA (R^2^ = 0.2078, *P* < 0.05), and negatively with LDFA (R^2^ = 0.2370, *P* < 0.05) (Fig. 4). Similarly, for mobile-bearing prostheses, postop HKA correlated positively with aHKA (R^2^ = 0.4605, *P* < 0.05) and MPTA (R^2^ = 0.223, *P* < 0.05), and negatively with LDFA (R^2^ = 0.2374, *P* < 0.05) (Fig. 5).

**Fig. 6:**
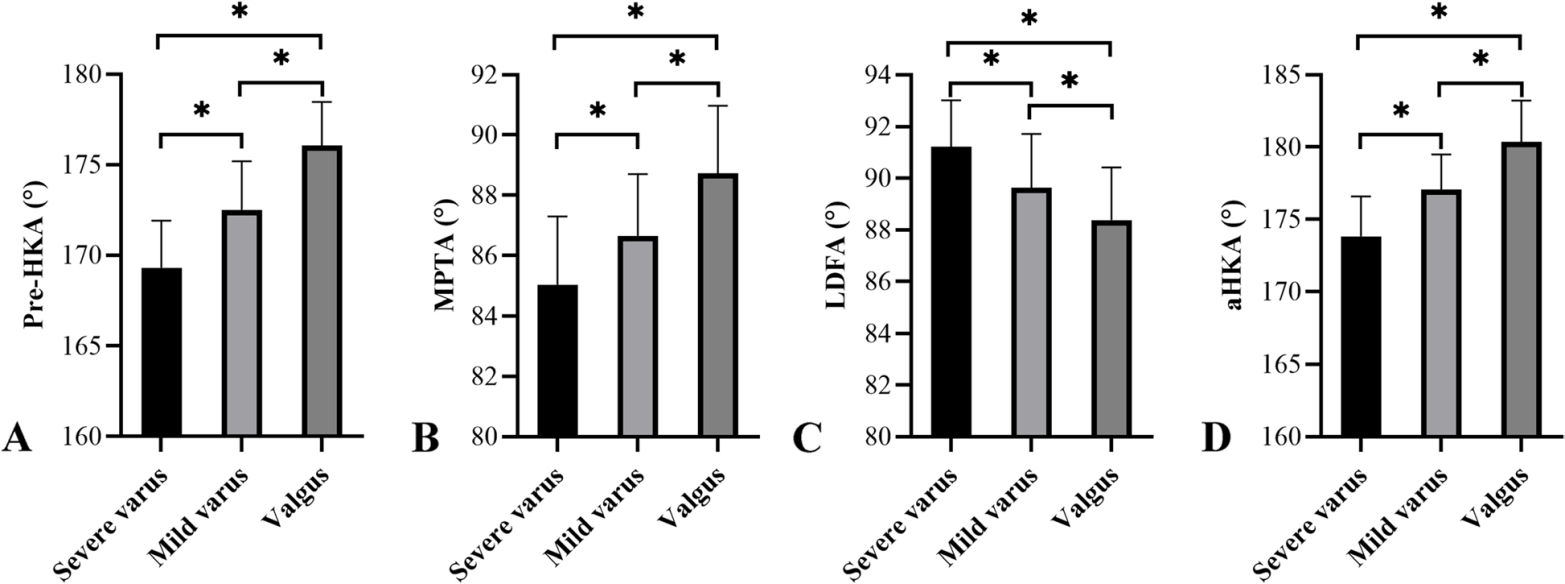
Mean and the standard deviation of pre-HKA (**A**), MPTA (**B**), LDFA (**C**), and aHKA (D) for each postop HKA angle group. Significant differences were found between each of the three groups (*P* < 0.05). HKA, hip-knee-ankle; MPTA, medial proximal tibial angle; LDFA, lateral distal femoral angle; aHKA, arithmetic hip-knee-ankle angle

Significant differences (*P* < 0.05) were observed among the valgus, mild varus, and severe varus groups for aHKA, MPTA, LDFA, and preoperative HKA. Higher postop HKA angles were associated with larger preoperative HKA, MPTA, and aHKA values, and smaller LDFA values. Conversely, lower postop HKA angles were associated with smaller preoperative HKA, MPTA, and aHKA values, and larger LDFA values. These findings align with the correlation analyses (Fig. 6).

## Discussion

The primary finding of this study was a significant positive correlation between the aHKA and postop HKA, with a correlation coefficient of R^2^ = 0.4595 (Fig. 4A). The aHKA (176.23° ± 3.15°) was closer to the postop HKA (176.09° ± 2.86°) than the preoperative HKA (171.71° ± 3.22°). Further analysis demonstrated that the MPTA also showed a significant positive correlation with postop HKA (R^2^ = 0.2072, *P* < 0.05), whereas the LDFA exhibited a significant negative correlation (R^2^ = 0.2448, *P* < 0.05). These relationships remained consistent for both fixed-bearing and mobile-bearing prostheses. Additionally, when stratified by postop HKA alignment (valgus, mild varus, severe varus), similar trends were observed for aHKA, MPTA, LDFA, and preoperative HKA across groups (Table 2).

**Table 2:** Radiological parameters in different prosthesis types

	Fixed-bearing (*n* = 161)	Mobile-bearing (*n* = 93)
Preoperative HKA (°)	171.98 ± 3.25	171.25 ± 3.13
MPTA (°)	86.28 ± 2.32	86.26 ± 2.38
LDFA (°)	89.88 ± 2.23	90.34 ± 2.04
aHKA (°)	176.40 ± 3.16	175.92 ± 3.13
Postop HKA (°)	176.46 ± 2.89	175.45 ± 2.70

HKA, hip-knee-ankle; MPTA, medial proximal tibial angle; LDFA, lateral distal femoral angle; aHKA, arithmetic hip-knee-ankle angle

While some studies suggest perioperative varus alignment may not affect short-term patient-reported outcomes in mobile-bearing UKA [21], preserving coronal alignment is associated with superior outcomes [22]. Suboptimal alignment risks complications, including component loosening and osteoarthritis progression, potentially leading to revision surgery [8, 23]. Biomechanically, postop varus alignment increases medial compartment stress, accelerating prosthesis wear and reducing longevity. Slaven et al. [9] compared 61 cases of aseptic loosening after medial fixed-bearing UKA with matched controls, finding significantly greater varus alignment in the loosening group (6.1° ± 3.1° vs. 4.0° ± 2.7°; *P* < 0.001). Similarly, Foissey et al. [24] reported increased tibial implant failure risk with joint line lowering and postop varus malalignment in a cohort of 366 UKAs (mean follow-up: 61.3 months).

Conversely, valgus malalignment from overcorrection is a common cause of lateral compartment osteoarthritis progression [25]. Xue et al. [26] observed that all cases of progressive lateral OA in their 708-knee cohort (follow-up > 12 years) exhibited overcorrection (postop HKA > 185°). Slaven et al. [8] also noted greater valgus alignment in patients with progressive lateral OA versus controls (0.3° ± 3.6° vs. 4.4° ± 2.6° varus; mean follow-up ≥ 10 years). These findings underscore the importance of mitigating risk factors for postop valgus malalignment. Notably, despite the surgeon's extensive experience at our center, 6.30% of cases (*n* = 16) developed postop valgus malalignment (Fig. 2C), highlighting the need to identify associated risk factors to optimize UKA outcomes.

In 1989, Kozinn and Scott [27] established patient selection criteria for UKA, including: preoperative range of motion ≥ 90°, flexion contracture < 5°, and angular deformity < 15°. For suitable UKA candidates, varus deformity typically stems from bone wear without intrinsic medial collateral ligament (MCL) contracture; the deformity should be passively correctable to neutral under valgus stress at 20° flexion [28]. The surgical principle involves restoring native MCL tension without ligament release. Consistent ligament tension theoretically ensures stable postop alignment, rendering the osteotomy angle irrelevant to overall limb alignment [29]. However, preoperative valgus-stress long-leg radiographs are rarely available, complicating accurate prediction of postop HKA. Furthermore, some surgeons intentionally select thicker polyethylene inserts to mitigate perceived risks of postop joint laxity, inadvertently increasing postop HKA. Therefore, standardizing surgical technique and developing reliable preoperative predictors of postop HKA are crucial.

aHKA has gained prominence for assessing constitutional lower limb alignment, particularly with the rise of kinematic alignment techniques in TKA that aim to restore pre-arthritic anatomy [30]. In early arthritis without significant bone loss, the LDFA and MPTA remain stable. Tarassoli et al. [16] found no significant difference between aHKA and stressed HKA measured via robotic navigation. Similarly, Mullaji et al. [31] and Kleeblad et al. [32] demonstrated that preoperative HKA predicts postop HKA. Recent studies further validate aHKA as a reliable alignment indicator. Liu et al. [33] reported that aHKA correlates with postop alignment in mobile-bearing UKA, with aHKA > 180° increasing valgus malalignment risk. Nakano et al. [20] likewise identified aHKA as a predictor of coronal malalignment in fixed-bearing UKA.

Our study utilized long-leg standing radiographs to measure pre- and postop HKA, LDFA, and MPTA. This method is widely accepted due to its simplicity and established correlation with CT-based measurements [34]. We observed a significant positive correlation between postop HKA and preoperative aHKA, consistent with Nakano et al. [20] and Zhang et al. [35]. Postop HKA also correlated positively with MPTA and negatively with LDFA. These relationships likely arise from: minimal soft tissue release during exposure, variable bone resection and insert thickness, and the assumption of pre- and postop joint line convergence angle (JLCA) = 0°. Under these conditions, postop HKA = MPTA − LDFA + 180°, equivalent to aHKA. Thus, a larger preoperative MPTA predicts a larger postop MPTA and HKA, while a larger preoperative LDFA predicts a smaller postop HKA. Previously, the lack of reliable predictors led to UKA in patients with aHKA > 180° despite preoperative HKA < 180°, resulting in postop valgus malalignment (observed incidence: 6.3% in this study). Incorporating preoperative aHKA assessment can improve patient selection (aHKA ≤ 180°), reduce valgus malalignment incidence, and potentially slow lateral compartment osteoarthritis progression.

This study has several limitations. Firstly, this retrospective study relied on long-leg standing radiographs. Despite efforts to standardize limb rotation between pre- and postop imaging, measurement errors remain possible. Secondly, preoperative valgus-stress radiographs were unavailable, and intraoperative osteotomy thickness was not recorded. Surgeon experiences guided insert selection, potentially leading to medial space discrepancies. Thirdly, we assessed the relationship between postop HKA and aHKA but did not evaluate the influence of tibiofemoral alignment following UKA. Lastly, focus was on overall limb alignment; prosthesis component alignment, which impacts postop HKA and clinical outcomes, was not measured. Future studies with long-term follow-up are needed to clarify relationships between postop prosthesis alignment, limb alignment, and clinical outcomes.

## Conclusions

In both fixed-bearing and mobile-bearing medial UKA, the aHKA correlated with the postop HKA. This finding suggests aHKA is a valuable predictor of postop coronal alignment in medial UKA.

## Data Availability

No datasets were generated or analysed during the current study.

## Abbreviations

UKA: Unicompartmental knee arthroplasty
aHKA: Arithmetic Hip-Knee-Ankle Angle
HKA: Hip-Knee-Ankle Angle
MPTA: Medial proximal tibial angle
LDFA: Lateral distal femoral angle
OA: Osteoarthritis
JLCA: Joint line convergence angle
